# Central Neurocytoma Treated Using Supratentorial Ventricle Radiotherapy: A Single-Institution Analysis of Five Cases in Adjuvant or Salvage Settings After Surgery

**DOI:** 10.7759/cureus.56132

**Published:** 2024-03-13

**Authors:** Takeshi Maemura, Atsuto Katano, Hirokazu Takami, Masashi Nomura, Shunsaku Takayanagi, Hideomi Yamashita

**Affiliations:** 1 Department of Radiology, The University of Tokyo Hospital, Tokyo, JPN; 2 Department of Neurosurgery, The University of Tokyo Hospital, Tokyo, JPN

**Keywords:** salvage therapy, adjuvant therapy, radiotherapy, supratentorial ventricle, central neurocytoma

## Abstract

Introduction: Central neurocytoma (CN) is an extremely rare tumor primarily located in the supratentorial ventricular system, categorized as a glioneuronal or neuronal tumor.

Methods: This study presented a retrospective analysis of five CN patients who received adjuvant or salvage radiotherapy. Patients, aged 31-59 years, underwent radiation doses ranging from 60 Gy to 50.4 Gy over 27-30 fractions.

Results: All patients achieved effective local tumor control without severe complications. The median follow-up period was 51.7 months, demonstrating 100% overall and progression-free survival rates.

Discussion: Our study's clinical outcomes align with previous research, despite the limitation of a small sample size. Emphasizing the necessity for additional research, our findings added to the potential evidence of radiotherapy in managing CN. Larger, long-term studies were needed to confirm these promising results.

## Introduction

Central neurocytoma (CN) is a rare entity that is classified as a glioneuronal or neuronal tumor (GNT) according to the 2021 World Health Organization (WHO) Classification of Tumors of the Central Nervous System [1]. The Central Brain Tumor Registry of the United States Statistical Report revealed that GNTs account for only 1.2% of intracranial tumors [2]. While most GNTs are benign, symptomatic cases can cause various problems, including headaches, epilepsy, nausea, and visual disturbances [3].

The CN is often located within the supratentorial ventricular system, specifically in the lateral and third ventricles [4]. Typical clinical symptoms include headache, nausea, vomiting, and gait disturbances due to increased intracranial pressure as they obstruct the intraventricular foramen [5]. In certain cases, patients may experience vision impairments and mental disturbances, which are prominent signs of this condition [6].

CN is characterized by an indolent growth pattern and low-grade histology and is classified as WHO grade II [7]. Li et al. reported immunohistochemical characteristics that revealed consistent expression of synaptophysin and neuron-specific enolase in many cases, whereas glial fibrillary acidic protein was present in only three of 15 cases [8]. They reported that the MIB-1 proliferation index varied from 0.8% to 12.5%. Kalawi et al. reported that methylation profiling can help differentiate neurocytomas from other tumors, thereby enhancing diagnostic accuracy [9].

Gross total resection (GTR) is the cornerstone treatment for CNs [10]. However, the incorporation of adjuvant or salvage radiotherapy in cases of subtotal resection (STR) or pathological malignancy remains a subject of continuous debate [11]. Balancing tumor control with the preservation of neurological function remains a significant challenge in the comprehensive management of CNs.

This study aimed to contribute to the knowledge of CNs by presenting the treatment outcomes of a single-institution cohort of patients who underwent adjuvant or salvage radiotherapy. We sought to provide valuable insights into the role of radiotherapy in managing CNs, including survival rates and treatment-related complications.

## Materials and methods

This study was a retrospective analysis of a single-institution experience involving patients diagnosed with CN who received adjuvant or salvage radiotherapy as part of their treatment. This study aimed to assess the treatment outcomes of these patients, with a specific focus on overall survival, progression-free survival, and treatment-related adverse events. This study included a cohort of five patients who were diagnosed with CN at our institution between June 2011 and November 2022. The inclusion criteria for this study were as follows: (i) histologically confirmed diagnosis of CN; (ii) received adjuvant or salvage radiotherapy following surgery; and (iii) availability of complete medical records and follow-up data. Patients who did not meet these criteria or had insufficient follow-up data were excluded. Data collection was conducted using electronic medical records, radiological imaging, and pathology reports of eligible patients. This study complied with the ethical standards outlined in the Declaration of Helsinki. The Research Ethics Committee of the Faculty of Medicine of the University of Tokyo obtained approval number 3372-6. The requirement for informed consent was waived owing to the retrospective nature of this study.

**Figure 1:**
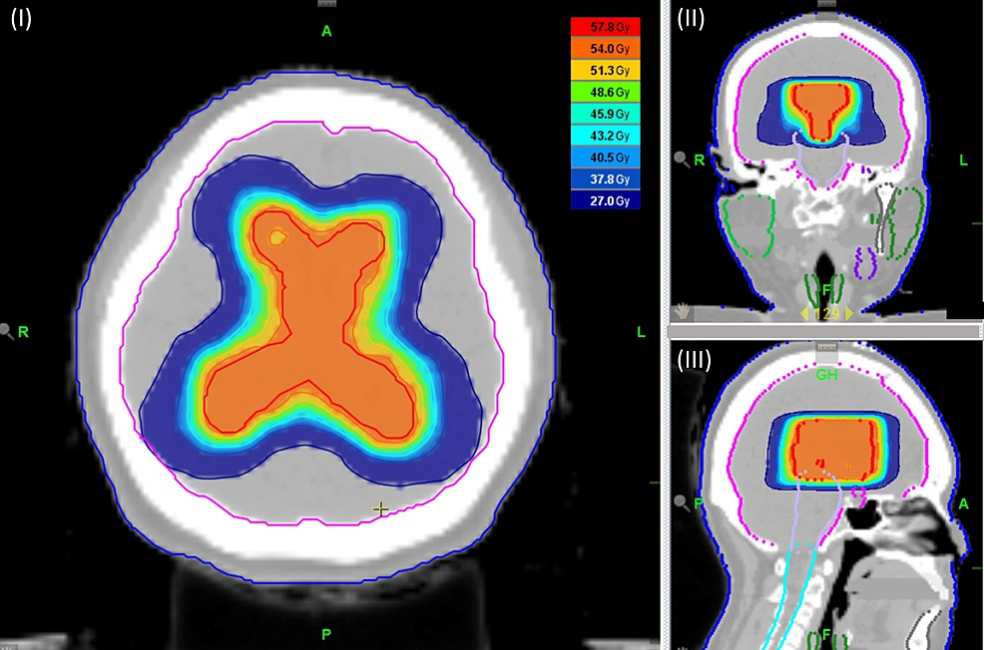
Dose distribution of radiotherapy Axial (I), sagittal (II), and coronal (III) dose wash images illustrating supratentorial ventricle radiotherapy with a dosage of 54 Gy in 27 fractions using helical tomotherapy

The radiation therapy protocols used in this study were as follows: The patient’s head was immobilized using a thermoplastic mask in the supine position during the planned computed tomography (CT). Both planning CT and magnetic resonance imaging (MRI) images were used for delineating the focal and whole ventricular target volumes. The clinical target volume (CTV) included residual or recurrent tumors, lateral ventricles, and the third ventricle as visualized on the T2 weighted image by MRI. The planning target volume on the radiotherapy plan was created by adding a 3-5 mm margin to the CTV to compensate for the deviation of the isodose curve due to geometrical uncertainties. In all cases, radiation therapy was performed by three-dimensional conformal radiotherapy or intensity-modulated radiotherapy (Figure 1).

Overall survival was defined as the length of time from the start of radiotherapy to death from any cause. Progression-free survival was defined as the time from the start of radiotherapy to either disease progression or death from any cause. The radiation dose and fractionation were determined by a radiation oncologist at the time of treatment.

## Results

The cohort consisted of five patients, including two males and three females with CN, who underwent adjuvant or salvage radiotherapy. The age at radiotherapy ranged from 31 to 59 years, with a median age of 34. The prescribed radiation doses varied between 60 Gy and 50.4 Gy, administered over 27-30 fractions. The median Karnofsky performance status (KPS) score at the initiation of radiotherapy was 100, ranging from 80 to 100 (Table 1).

**Table 1:** Patient characteristics (n=5) KPS: Karnofsky performance status, RT: radiotherapy, GTR: gross total resection, STR: subtotal resection, N.E.R: no evidence of recurrence

Age (years)	Sex	KPS	MIB-1 index	Extent of resection	Aim of the RT	Interval of surgery to RT (months)	Radiation dose (Gy)	Follow-up duration after RT (months)	State
31	F	100	2%	GTR	Salvage	17.5	60 Gy/30 fr	89.6	N.E.R
31	F	90	4%	STR	Salvage	22.7	60 Gy/30 fr	118.1	N.E.R
36	M	100	3%	STR	Adjuvant	2.5	60 Gy/30 fr	22.2	N.E.R
34	F	80	10%	GTR	Adjuvant	0.7	54 Gy/27 fr	51.7	N.E.R
59	M	100	10%	STR	Adjuvant	1.6	50.4 Gy/28 fr	10.6	N.E.R

All five patients in this study exhibited effective local tumor control following adjuvant or salvage radiotherapy. Post-treatment imaging, including MRI, indicated a stable or reduced tumor size in all cases.

The median follow-up duration was 51.7 months (range: 10.6-118 months). Overall and progression-free survival rates at the end of the follow-up period were 100%. The adverse events associated with radiotherapy were mild and manageable. Acute phase side effects included fatigue (three patients), mild headache (three patients), and alopecia (two patients). No severe complications or treatment-related mortality were observed in the late phase.

## Discussion

The results of this single-institution study demonstrated that adjuvant or salvage radiotherapy is an effective treatment modality for CNs. It provides excellent local tumor control, symptom relief, and a high survival rate without significant treatment-related complications. These findings support the role of radiotherapy as a valuable adjunct to surgery in the management of CN.

Samhouri et al. analyzed 33 CN patients who underwent surgery in an international multicenter study and found a five-year overall survival rate of 90% and a progression-free survival rate of 76% [12]. Among them, 19 patients received radiotherapy with a median radiation dose of 54 Gy (range: 50-60 Gy), leading to a significantly longer progression-free survival. This study emphasized that radiotherapy enhances survival rates in CN patients while maintaining manageable side effects. Chen et al. examined 63 CN patients who received adjuvant radiotherapy after surgery [13]. Adjuvant radiotherapy after incomplete resection demonstrated outcomes comparable to those after complete resection. Zhang et al. examined 413 CN patients using the surveillance, epidemiology, and end results database and did not recommend radiotherapy after GTR [14]. Imber et al. analyzed 28 CN patients and reported a two-year progression-free survival of 75%. MIB-1 labeling >4% predicted poorer outcomes, and their data suggested that adjuvant radiotherapy after STR may improve progression-free survival [15].

Stereotactic radiosurgery (SRS) has recently been considered an alternative surgical approach for intracranial benign tumors [16-19]. Several studies have reported favorable outcomes for CN treated with SRS. Hung et al. conducted a study of 60 CN patients treated with SRS and reported excellent tumor control (93% at five years) and manageable complications [20]. Park et al. conducted a systematic review of the available data on SRS for CN by analyzing 35 eligible studies [21]. The results showed a cumulative rate of tumor control of 91.1% (95% CI = 80.2-96.3%) after a mean follow-up of 59.3 months (range 6-140 months).

It is important to note that this study is limited by its small sample size. Further research with a larger patient population is warranted to confirm these promising results. Long-term follow-up and multi-institutional studies may provide additional insights into the long-term efficacy and safety of adjuvant or salvage radiotherapy for CN. It should also be emphasized that CN can occur in the fourth ventricle, although rarely [22,23].

## Conclusions

Based on the outcomes observed in this study, adjuvant or salvage radiotherapy appears to be a beneficial treatment option for CN, offering favorable tumor control rates and a high overall survival rate with limited adverse events. In this study, adjuvant radiotherapy was recommended for patients with a high likelihood of recurrence, such as a high MIB1 index, whereas salvage therapy was selected as a treatment strategy for those who experience tumor progression. The choice between adjuvant and salvage therapies should be made with careful consideration of individual patient factors.
